# RNA Secondary Structure as a First Step for Rational Design of the Oligonucleotides towards Inhibition of Influenza A Virus Replication

**DOI:** 10.3390/pathogens9110925

**Published:** 2020-11-07

**Authors:** Marta Szabat, Dagny Lorent, Tomasz Czapik, Maria Tomaszewska, Elzbieta Kierzek, Ryszard Kierzek

**Affiliations:** Institute of Bioorganic Chemistry, Polish Academy of Sciences, Noskowskiego 12/14, 61-704 Poznan, Poland; dlorent@ibch.poznan.pl (D.L.); tczapik@ibch.poznan.pl (T.C.); mtomaszewska@ibch.poznan.pl (M.T.); Elzbieta.Kierzek@ibch.poznan.pl (E.K.)

**Keywords:** influenza A virus, RNA structure, replication, antiviral strategies, RNA interference, antisense oligonucleotides, catalytic nucleic acids

## Abstract

Influenza is an important research subject around the world because of its threat to humanity. Influenza A virus (IAV) causes seasonal epidemics and sporadic, but dangerous pandemics. A rapid antigen changes and recombination of the viral RNA genome contribute to the reduced effectiveness of vaccination and anti-influenza drugs. Hence, there is a necessity to develop new antiviral drugs and strategies to limit the influenza spread. IAV is a single-stranded negative sense RNA virus with a genome (viral RNA—vRNA) consisting of eight segments. Segments within influenza virion are assembled into viral ribonucleoprotein (vRNP) complexes that are independent transcription-replication units. Each step in the influenza life cycle is regulated by the RNA and is dependent on its interplay and dynamics. Therefore, viral RNA can be a proper target to design novel therapeutics. Here, we briefly described examples of anti-influenza strategies based on the antisense oligonucleotide (ASO), small interfering RNA (siRNA), microRNA (miRNA) and catalytic nucleic acids. In particular we focused on the vRNA structure-function relationship as well as presented the advantages of using secondary structure information in predicting therapeutic targets and the potential future of this field.

## 1. Introduction

Influenza is a significant research subject for scientists around the world due to its serious threat to public health. Generally, influenza A virus (IAV) is a human and animal pathogen causing seasonal epidemics and sporadic pandemics resulting in high morbidity and mortality. It is estimated that due to the Spanish flu in 1918/1919 up to 100 million people died [1]. The pandemic potential of influenza virus is a result of its high genome variability and is correlated with its pathogenicity, replication, growth kinetics as well as the viral life cycle including replication dynamics, RNA packaging, RNA editing and mRNA splicing. These processes can be controlled by the virus RNA structure [2].

The IAV belongs to the Orthomyxoviridae family of enveloped viruses. Its genome consists of eight negative-sense viral RNA (vRNA) segments that encode for 11 proteins including glycoprotein hemagglutinin (HA) and neuraminidase (NA), a nucleoprotein (NP), two matrix proteins (M1 and M2), three polymerase complex proteins: polymerase basic protein 1 (PB1), polymerase basic protein 2 (PB2) and polymerase acidic protein (PA) as well as four non-structural proteins (NS1, NS2, PA-X and PB1-F2) [3]. HA and NA proteins predominantly mediate viral entry and release of the progeny of the virus from an infected host cell. Activation of the viral HA by a host cell protease is essential for viral infectivity. Genes encoding for HA and NA are important segments in a process called antigenic drift, leading to point mutations in these genes, and antigenic shift, which is a reassortment of the segments. These two kinds of genetic variation create new strains or subtypes of the IAV [4,5]. On the contrary, viral polymerase proteins (PA, PB1, PB2) and NP are more conservative and responsible for influenza virus replication. M1 protein forms a coat inside the viral envelope and binds to vRNA, whereas M2 is an ion channel which governs virus uncoating and assembly [4]. 

The vRNA is replicated into positive-sense complementary RNA (cRNA) and transcribed into viral messenger RNA (mRNA) inside infected host cells. The vRNA and the cRNA are shielded with viral NP. In viral particles, the vRNA is associated with the polymerase, consisting of three subunits: PB1, PB2 and PA [6]. The vRNA with multiple NP monomers and polymerase subunits forms the viral ribonucleoprotein complex (vRNP), which is an independent transcription-replication unit. Upon viral infection, the vRNP is transported into the nucleus of the host cell, where transcription and replication of the viral genome occur. Next, vRNP is exported, assembled and budded at the host cell plasma membrane [7,8]. RNA is used throughout the life cycle, highlighting its fundamental role in influenza biology.

The viral RNA is involved in many processes through activities which relate to its secondary and tertiary structures. The correct packaging of all eight vRNA segments into one viral particle is suggested to be conferred at least partially via structured RNA motifs of vRNA protruding from vRNP. Within the viral particles, the vRNPs present a highly flexible double helical structure, with the polymerase at one end and a closing loop at the other [6]. It is known that vRNA and mRNA form complex secondary structures that are conservative among influenza A virus strains [2]. Additionally, it has been shown that different secondary structures of the vRNA at various stages of the viral life cycle can contribute to replication and transcription regulation. The role of some structural motifs of viral RNA has been confirmed experimentally [9,10,11,12,13]. Due to the fact that vRNP is an independent transcription and replication unit and that the viral RNA plays an important role in packing of progeny virus, vRNA can be an interesting therapeutic target.

The segmented nature of IAV RNA serves as a good target for potential new antiviral strategies. Due to the high genetic variability of the influenza virus and the ease of its transmission, protective vaccination and anti-influenza drugs currently used are not fully effective. Therefore, there is a necessity to search for new approaches to limit the influenza spread. These days, the only anti-influenza drugs approved by the FDA are small ligands that bind to the surface of glycoprotein neuraminidase. Previously used ion channel M2 inhibitors are no longer recommended in therapy due to the emergence of the resistant strains [14]. 

Antigenic shift and drift as well as adapting mutations allow the influenza virus to effectively evade host immune response. Since the variable nature of the influenza virus makes development of an effective vaccine challenging, its formula must be changed seasonally. Moreover, it cannot provide protection against future pandemic strains [15]. This suggests that novel therapeutic approaches with higher specificity for a broader range of strains and/or combination of alternative strategies to reduce the likelihood of resistance are needed.

The design of currently used therapeutics is based on the prediction of a three-dimensional structure of viral proteins and interactions between potential inhibitor and protein(s). Intensive crystallography, cryo-EM and computational studies have provided new insights into secondary, tertiary and quaternary structure of the influenza proteins and the potential inhibitory small molecule ligands [16,17,18]. However, the prediction of its stability and the modulatory effect of other proteins remain unclear and require further research.

As an alternative, advances in primary and secondary RNA structure determination have led to the development of novel and effective anti-influenza agents. RNA-based approaches can potentially restrain spreading of the infection by interacting with vRNA, cRNA or mRNA [9,10,19]. To control and silence gene expression, synthetic oligonucleotides, including antisense oligonucleotides (ASOs), small interfering RNAs (siRNAs) and microRNAs (miRNAs) as well as catalytic nucleic acids, have been broadly investigated. Their mechanism of action is based on selective binding to complementary sequences of target influenza RNAs. Nowadays, oligonucleotides carry several chemical modifications. It increases their nuclease resistance and thermal stability as well as binding affinity to influenza target RNAs [20,21]. In this review, we will briefly describe ASOs, siRNAs, miRNAs and catalytic nucleic acids as anti-influenza tools and approaches with a particular focus on the viral RNA structure-function relationship. We will discuss the implications of using influenza RNA secondary structure information in therapeutic targets prediction and the potential future of this field.

### 1.1. Anti-Influenza Strategies Based on Oligonucleotide Tools: ASOs, siRNAs, miRNAs and Catalytic Nucleic Acids Targeting Conserved Viral RNA Regions

Sequence and structural motifs of influenza RNAs are partially conserved between strains due to the combination of evolutionary constrains. Therefore, various oligonucleotide tools are designed to alter RNA expression and function, thus allowing to influence processes beyond protein or receptor involvement [22,23]. They include, for instance, the usage of antisense oligonucleotides or RNA interference approaches that target the most conserved regions of viral RNA. These strategies are based on the sequence complementarity between the target RNA and ASO/miRNA/siRNA, which leads to the inhibition of target gene expression by either a steric block in translation, mRNA degradation or both [23]. In this section we will discuss the examples of influenza inhibition experiments performed using ASOs, siRNAs, miRNAs and catalytic nucleic acids designed in a traditional way based on target sequence analysis, and we will focus on various approaches to enhance their effectiveness.

#### 1.1.1. Antisense Oligonucleotides

Antisense oligonucleotides (ASOs) are synthetic, single stranded, short (12–30 nucleotides long) DNA or RNA fragments. They selectively bind to complementary regions and block target genes by several different mechanisms. Based on their mode of action, ASOs can be divided into three main groups: steric blockers of translation, modulators of splicing and recruiters of protein factors (e.g., RNase H) that lead to the degradation of target RNA. The functions of target RNA as well as the selected interaction site determine the modulatory effect of ASOs [20]. Thus, the design of specific ASOs should be preceded by prediction of RNA secondary structure, which allows identifying local structures and conserved motifs accessible to ASO hybridization. Many factors can affect the activity of ASOs on their intended RNA targets. The strength of the therapeutic effect of a given ASO in cells will also depend on its pharmacokinetics, toxicity and delivery method [24]. Nevertheless, since information about nucleotide sequence of targeted RNA is sufficient to design ASO, the application of antisense nucleotide technology has been investigated in potential influenza therapies for many years. Changes in viral proliferation upon ASO binding were tested not only in cell cultures, but also in model organisms such as mouse and chicken [25,26,27,28,29]. Generally, ASOs activity is improved considerably when it contains modified nucleotide residues and is delivered as a conjugate with different ligands or nanocarriers [28,30,31].

Liposomal encapsulation was used to deliver ASOs binding to functionally important sequences of packaging signals at the 5′-ends of PA and PB1 segments [25]. Although intended interacting sites had overlapping fragments, ASOs exhibited different antiviral activity. Notably, the highest decline in virus titer was noted for ASOs targeting analogous regions of PB1 and PA [25]. These findings highlight the importance of the structure and function of target RNA studies when designing ASO tools. In another study, Levina et al. constructed a nanocomposite of ASO targeting NP 3′-end of the vRNA segment not covalently bound to TiO_2_ nanoparticles, which allowed a complete replication inhibition of different influenza subtypes in MDCK cells [31,32]. In contrast to other recent research carried out in this area, DNA sequences of ASOs were unmodified. Due to the nanotechnology-based delivery method, they exhibited a greater antiviral effect even at two orders of magnitude lower concentration [31,32] in comparison to previously tested morpholino ASOs binding in the same region [27].

Currently, most antisense strategies involve particular ASO binding only to one target in the viral genome. Innovative approach was investigated by Duan et al., who designed the universal phosphorothioate antisense oligonucleotide, specific for the 5′-terminal conserved sequence found in all eight vRNA segments of the influenza virus [26]. In MDCK cells, ASOs caused an inhibitory effect against influenza viruses belonging to different strains. When tested in IAV-infected mice, the protective effect of ASO did not improve survival of the treated animals [26]. In 2017, in order to improve the bioavailability of tested ASO, a targeted delivery system based on peptide with high affinity to influenza HA was developed [28]. ASO conjugated to an HA-specific peptide, which effectively entered in in vitro cell culture as well as mice lungs and significantly inhibited viral replication. When a conjugated molecule was intranasally administrated to IAV-infected mice, 80% of treated animals survived infection [28]. This means that the delivery approach based on chimeric peptide-ASO conjugates can improve cellular uptake of the therapeutic ASO tool. 

Another specific delivery strategy in influenza treatment was developed by Zhang et al., who conjugated single chain variable fragment (scFv) directed against influenza HA antigen with truncated protamine (tP) [30,33]. ASOs designed in this study were phosphorothioate-modified analogues targeting coding, and untranslated regions in the PA segment involved in cRNA promoter binding activities of RNA polymerase. All tested ASOs inhibited the replication of the influenza virus in MDCK cells. Interestingly, ASOs targeting coding regions decreased viral titer, whereas ASOs interacting within the untranslated site exhibited lower antiviral activity. To verify if the antibody-mediated delivery system can enhance therapeutic effect, the most potent ASO, called PA492, was conjugated to fusion protein scFv-tP and then tested in cell culture and mouse model. ScFv-tP-mediated PA492 showed a higher antiviral activity against influenza virus than liposome-mediated PA492. Injection of fusion protein-mediated PA492 into IAV-infected mice resulted in 63% survival of mice, while for liposome-delivered PA492 the rate was 50%. Importantly, in vivo tests with fluorescein isothiocyanate labeled-PA492 (FITC-PA492) revealed that ScFv-tP-mediated delivery system specifically directed ASO to the lungs. On the contrary, liposome-delivered FITC-PA492 was mainly observed in the liver, spleen and kidney [30,33,34]. Described results demonstrating high effectiveness of delivery system made up of anti-HA fusion protein and ASO provides a powerful methodology for targeted drug delivery in influenza treatment.

Thus far, most studies on the ASO strategy towards the inhibition of the influenza proliferation have been reported as successful in both cell culture and animal models. Radavirsen (AVI-7100) was the first and currently only ASO in influenza treatment that entered clinical trials. Radavirsen is a 20-mer PMOplus®-based ASO, designed to bind highly conserved regions responsible for splicing M1 and M2 transcripts of M (+)RNA segment. The initial experiments in preclinical ferret, mouse, rat and monkey models revealed the antiviral activity of ASO and allowed to assess its toxicity. Next, a single and multiple ascending dose phase I clinical study was conducted in healthy volunteers. Since Radavirsen was well tolerated with no safety concerns identified, the results obtained by Beigel et al. are promising for the use of oligonucleotide drugs in influenza therapy [35,36].

Recent advances in ASO chemistry and delivery methods have led to the discovery of various promising candidates for influenza treatment. Remarkably, preventing viral proliferation with the ASO strategy has been achieved by targeting other elements than influenza RNA segments, including host-encoded hemagglutinin-activating protease TMPRSS2 [37] and lymphocyte activation gene-3 [38]. However, the reasons for the inefficiency of some ASOs are not yet entirely understood. It seems that the rational prediction of ASO binding sites with respect to target viral RNA structures, accessibility of complementary regions and their function in viral proliferation may provide a strategy for successful ASO design and therapeutic applications. 

#### 1.1.2. Small Interfering RNA 

Small interfering RNA (siRNA) is an oligonucleotide approximately 21–25 nt long formed through intracellular Dicer enzyme cleavage of the excises of exogenous double-stranded RNA. The siRNA induces sequence-specific degradation of targeted mRNA and can specifically inhibit the replication of influenza virus [39]. In general, siRNA cleavage action depends on its specific features [40]. The siRNA tools have been widely used in the research of anti-influenza viral infection, because they have high specificity and selectivity as well as silencing efficiency. 

It has been shown that synthetic siRNAs can specifically silence the influenza virus mRNA and inhibit the virus replication at different levels [41]. Ge et al. revealed that specific siRNA induced RNA interference (RNAi) by directly targeting viral mRNA for degradation, and indirectly inhibiting all viral RNA transcription [41]. The researchers showed that siRNAs potently inhibited influenza virus production in both cell lines and embryonated chicken eggs. Interestingly, they observed that siRNA specific for NP or PA interfered with the accumulation of not only the corresponding mRNA but also NP- or PA-specific cRNA and vRNA. Designed siRNAs eliminated the accumulation of other viral, but not cellular, RNAs. This study has contributed to the development of antiviral siRNA drugs by targeting the conserved regions of mRNA sequences and potentially inhibiting the IAV replication in vitro. In 2015, Jain et al. designed siRNAs that targeted the most conserved region of the M gene present in the prevalent IAV strains (delineated by aligning multiple sequences of human pathogenic IAV submitted to the NCBI database) [42]. The ORF of the M gene was targeted by the designed siRNA and its efficiency in vitro was tested using two IAV strains (H1N1 and H3N2). The researchers observed the inhibitory effect of siRNA at 48 h post infection on the MDCK cell line. Their study showed that the inhibition of the replication of influenza A H1N1 and H3N2 strains was 74% and 62%, respectively [42]. 

An interesting observation was made by McMillen et al., who described that the NS1 gene-specific siRNAs caused decreasing mRNA level, while in case of siRNAs targeting NA and M genes, mRNA and protein expression levels were upregulated in culture lysates [43]. This can suggest that defective influenza virus may have been produced during the RNAi process. The researchers designed a number of effective influenza-targeting siRNAs and studied their effect against IAV infection in vitro. The results revealed that most tested siRNAs attenuated influenza virus replication. However, some designed siRNAs reduced viral RNA levels and both NS1 and NS2 expression in culture lysates, but did not inhibit viral replication. Moreover, this study also showed that the influenza virus titer decreased and that the combination siRNA treatment can lead to a more potent attenuation of influenza infection than single siRNA treatment [43]. In another study, Huang et al. presented the replication inhibition by RNAi targeting various genes of IAV in vitro and in a mouse model by intratracheal delivery [39]. They prepared a series of DNA vector-based short hairpin RNAs (shRNAs) which targeted the IAV genes encoding the PA and PB2. The authors showed that three of these shRNAs efficiently inhibit IAV replication in vitro. Furthermore, they found that PB2-targeting shRNA can effectively reduce the mortality rate of the mice after influenza virus infection. Their investigations revealed that the PB2-targeting shRNA plasmid can be a potential RNAi-based tool for influenza virus infection [39]. 

Recently, it has been demonstrated that siRNA molecules can efficiently inhibit the replication of human seasonal and avian influenza viruses in various types of human cell systems [44]. Jiang et al. generated a series of siRNAs using enzymatically produced IAV-specific dsRNA and *Giardia intestinalis* Dicer and evaluated the efficacy of these molecules in preventing viral infection in human primary monocyte-derived macrophages and dendritic cells. These siRNAs contained more than 100 different antiviral RNAs targeting the most conserved regions of the IAV genome. Results revealed that the replication of different IAV strains was significantly inhibited by IAV-specific siRNAs. The authors observed an increase in inhibition of viral RNA expression by up to seven orders of magnitude and a reduction of IAV protein synthesis as well as virus production. This research showed that the wide antiviral spectrum makes the siRNA a potentially efficient tool against multiple strains of influenza virus, acting by targeting their conserved gene regions [44]. 

Interesting research has been conducted by the Vasin group, who developed a highly effective hybrid microcarrier system for the delivery of antiviral siRNA mixtures [45]. They used a series of novel siRNAs, targeting the most conserved fragments of three IAV genes, i.e., NP, PA and NS, and designed hybrid microcarriers containing a cocktail of siRNAs. They presented that this system can help to overcome extracellular and intracellular barriers, while protecting encapsulated genetic material. They also demonstrated the low toxicity of these hybrid microcarriers in vitro. Results showed that the siRNAs loaded into hybrid microcarriers more efficiently inhibited the replication in several IAV subtypes in comparison with single siRNA treatment. The researchers developed hybrid microcarrier technology containing a therapeutic siRNA mixture, which can be a promising anti-influenza approach [45]. 

To summarize, on the one hand, the siRNAs have high specificity, strong selectivity and silencing efficiency. Therefore, these molecules have been developed and used in the studies as antiviral tools against influenza, including highly pathogenic and avian influenza virus strains. On the other hand, siRNAs can be responsible for several problems, resulting in off-target effects. Hence, the current studies of the anti-influenza approach based on the siRNA-mediated RNAi process are limited to cells, chick embryos and animals and have not yet entered clinical trials.

#### 1.1.3. MicroRNAs

MicroRNAs (miRNAs) are small noncoding RNAs that control the translation and transcription of its target genes. These molecules can also regulate various biological processes, including viral infection. Emerging studies indicate that the host miRNAs play an important role in the intricate host-pathogen interaction network [46,47]. Cellular miRNAs are involved in antiviral responses and can directly target viruses and inhibit their infection. Additionally, viral infection is an important factor in driving the differential expression of miRNAs [48]. Therefore, these small endogenous molecules may serve as a therapeutic strategy. Moreover, the delivery of exogenous miRNAs can gain potential interest in antiviral therapy.

The Kumar group presented that the miRNA-485 (miR-485) can regulate antiviral immunity and restrict viral replication via targeting host and influenza virus transcripts [46]. They demonstrated that upon viral infection the cytosolic sensor of viral RNA (retinoic acid-inducible gene I, RIG-I) was activated and the miR-485 expression increased. Next, miR-485 directly targeted the 3′-UTR of the gene encoding RIG-I and induced degradation of RIG-I mRNA. As a consequence, the antiviral response was suppressed and the viral replication enhanced. The results also revealed that miR-485 can bind to a conserved region in the PB1 gene of IAV in sequence-specific manner and inhibit viral replication. These findings indicate the dual role of miR-485 in the regulation of antiviral signaling and the restriction of influenza virus infection [46]. 

In 2017, Peng et al. reported that the endogenous miRNAs mediate antiviral defense against IAV [49]. They screened approximately 300 miRNAs from human and mouse epithelial cells and studied their inhibitory effect in vitro and in vivo. Results identified five miRNAs that efficiently inhibited influenza virus replication. Moreover, these studies suggest that miRNAs are involved in the different stages of the viral life cycle regulation via targeting either viral RNA segments or the supportive host factors in antiviral defense [49]. Recently, Zhao et al. reported that endogenous miRNA-340 (miR-340) can regulate both antiviral responses and infection with RNA viruses [50]. The researchers found out that the viral NP, M1 and PA mRNAs levels noticeably increased in miR-340 overexpressing cells, while the inhibition or knockdown of miR-340 significantly inhibited virus replication. Furthermore, data from next-generation sequencing revealed that miR-340 can attenuate cellular antiviral immunity, and its suppression clearly prevents the infection of cells. These studies highlight the role of miR-340 as a potential regulatory factor involved in virus-host interactions [50]. 

Previous studies revealed a novel antiviral miRNA targeting the NS1 of the pandemic human influenza virus strains [51]. Bavagnoli et al. identified the human miRNA (hsa-miR-1307-3p) as a potent suppressor of NS1 expression, and consequently, IAV replication. Moreover, results from transcriptomic analysis revealed that this miRNA can also negatively regulate apoptosis and promote cell proliferation. In addition, the researchers identified the nucleotide substitution in the NS1 gene of the IAV strains, which is at the putative target site of the hsa-miR-1307-3p. They observed that this mutation in the NS1 enabled the influenza virus to escape the miRNA inhibition, giving replicative advantage to the virus in human cells. The obtained results allowed identifying both the target sequence in the NS1 gene and the corresponding antiviral miRNA; thus, it might be a starting point for the development of novel antiviral therapies [51]. In another study, it has been established that a broad spectrum anti-IAV miRNA screening allowed to identify five cellular miRNAs universally targeting PB1, PB2, PA or NP of the influenza A virus [52]. Next, the antiviral effectiveness of these miRNAs at both mRNA and protein levels was determined. The results showed that miR-188-3p could effectively inhibit the replication in infected cells by targeting PB2 mRNA. This miRNA downregulated PB2 expression at both mRNA and protein levels, which suggested that it may be used in RNAi-mediated antiviral therapeutic strategy [52].

Apart from the miRNA-based tools aimed at the target sequence of IAV genome, this approach can also be used to enhance biosafety of gain-of-function IAV research. For instance, tenOever’s group described a species-specific attenuation strategy, which allowed to restrict transmission in one species while maintaining transmission in the other hosts [53]. The authors used endogenous miRNA-192 (miR-192), expressed in human A549 lung cells but absent in ferret lung cells, and engineered an IAV strain that contained miR-192 target sites within the ORF of the HA segment. Replication of IAV with miR-192 target sites was abrogated in human cells, in contrast to the ferret model [53]. It suggested that an miRNA-based approach could be used to design or develop live attenuated influenza vaccines. 

Collectively, the examples of the studies described above indicate that host cells can lower their viral loads by controlling miRNA pathways, which may provide new opportunities for anti-influenza approaches. Investigation of miRNA molecules that directly target the conservative viral sequences can lead to the discovery of new therapeutics against influenza virus infection.

#### 1.1.4. Catalytic Nucleic Acids

Ribozymes and deoxyribozymes are two types of nucleic acids that have catalytic activity. Ribozymes have been shown to play an important role in biological processes such as splicing, RNA processing and replication of RNA genomes [54,55]. Some ribozymes occurring in nature are able to cleave the phosphodiester bonds of nucleic acids. Their deoxyribonucleic counterparts have not yet been found in nature. However, there are known synthetic DNAs that cleave RNA strands. The well-established examples are hammerhead and hairpin ribozymes, which form the classes of small size catalytic nucleic acids. The hammerhead ribozyme has a conserved catalytic core flanked by two arms complementary to the target RNA. The cleavage site is recognized by the NUH triplet sequence (N—any nucleoside, H—any nucleoside except guanosine) [56]. The hairpin ribozyme is a structural motif that includes two domains made of two short helices separated with an internal loop. One domain is responsible for substrate recognition and the other catalyzes the cleavage of the substrate [56]. 

DNA enzymes, also known as DNAzymes, were identified using in vitro selection methodology [57]. Two DNAzymes, named 10-23 and 8-17, have catalytic activity and show divalent cation-dependent RNA cleavage. The 10-23 DNAzyme is shown to cleave all purine-pyrimidine dinucleotide junctions, and the 8-17 DNAzyme cleaves RNA substrate between adenosine and guanosine, and requires a G-T wobble pair located after the cleavage site [58]. In general, DNA enzymes can be made to cleave different RNA substrates efficiently and specifically. Thus, such molecules can be used to inhibit any RNA target and can be developed as therapeutic agents. For instance, the ability of nucleic acid to catalyze the cleavage of RNA can be utilized to reduce viral replication. Tang et al. showed that IAV replication can be inhibited using ribozyme tools in both in vitro and in vivo tests [59]. The authors designed and characterized hammerhead and hairpin ribozymes that efficiently cleaved vRNA segment 5 of IAV. Based on the in vitro results the hammerhead ribozyme was considerably more efficient compared to the hairpin. Moreover, the catalytic activity of two ribozymes was also investigated under in vivo conditions. It was observed that the level of cellular resistance to IAV strain infection was up to 70–80% when cells were transfected with ribozyme-expression plasmids [59]. In another study, Lazarev et al. used the hammerhead ribozyme to inhibit IAV replication by targeting PB1 mRNA [60]. The researchers designed and prepared a construct used for the permanent expression of the ribozyme in cell lines. After cell infection with IAV strains, namely, A/Singapore/1/57 and A/WSN/33, the reduction of the virus reproduction and virus-specific protein synthesis were investigated. It was found that the maximum inhibition level of virus reproduction in transformed cells reached 94%. Moreover, this study revealed that the inhibition level of the NP and NS1 proteins’ synthesis in the cell line expressing ribozyme was 91% and 89%, respectively [60]. 

Interesting study by Kumar et al. presented a new approach by constructing siRNA-ribozyme chimera as a potential inhibitor for influenza virus [61]. The designed chimeric construct targeted the M1 genome segment of IAV. The researchers prepared wild-type siRNA-ribozyme construct and its sequentially mutated variants. The results showed that unmodified siRNA-ribozyme chimera reduced the expression of M1 gene by up to 67%. However, when the siRNA part of this chimeric construct was mutated, it resulted in reduction of the inhibition level to 33%. On the contrary, when the ribozyme part was catalytically inactive, keeping the siRNA unchanged, the observed inhibitory effect was about 20% [61]. In a subsequent study, researchers targeted the same M1 gene of IAV using designed ribozymes and DNAzymes [62]. These tools were tested and showed insignificant inhibition of influenza virus replication, but their effect was enhanced by the simultaneous use of both catalytic nucleic acids. Furthermore, the authors also investigated the cleavage potential of ribozyme in the presence of antisense oligonucleotide designed to hybridize upstream or downstream of tested ribozyme. This study revealed more efficient inhibition of M1 gene expression using ribozyme with addition of ASO. It shows that antisense oligonucleotide enhanced the ribozyme-mediated cleavage [62]. 

Motard et al. engineered ribozyme construct based on the hepatitis delta virus (HDV) and a specific on/off adapter (SOFA), which increases the specificity of the cleavage while reducing the non-specific effect [63]. They proposed a step-by-step selection strategy of the ribozymes, cleaving conserved regions of the influenza A subtypes. Next, the researchers used the most promising candidates of SOFA-HDV ribozymes to catalytically cleave the corresponding viral mRNA targets. They conducted catalytic activity assays in vitro as well as tested antiviral activity in cell culture and in vivo. It was reported that only one SOFA-HDV ribozyme combination significantly inhibited virus replication, with up to 1000-fold reduction in viral load in lung tissue cells [63]. 

In another study by Toyoda et al. reported the usage of 10-23 DNAzymes targeting the AUG initiation codon of PB2 mRNA of IAV [64]. They prepared DNAzymes and tested their RNA-cleaving activity under in vitro conditions. The investigations showed that DNAzymes inhibited viral replication in cultured cells more effectively than the same amount of ASO. However, the cleavage potential of DNAzymes was found to be low under physiological conditions. Additionally, the results revealed that DNAzymes with longer arms and complementary to the target region showed higher cleavage activity than those with shorter arms. Moreover, the longer DNAzyme led to a more efficient virus replication inhibition [64]. Another approach for the same target influenza A/PR8/34 PB2 mRNA initiation codon AUG was studied by Takahashi et al. [65]. The authors reported two 10-23 DNAzymes, named PB2Dz-9-N and PB2Dz-9-N(*2), which were modified on 3′- and 5′-termini with N3′-P5′ phosphoramidate bonds, which significantly enhanced their nuclease resistance. From the results, one can conclude that such modifications did not influence the efficacy of DNAzymes. The in vitro cleavage activity of these modified DNAzymes was the same as for the unmodified variants. Moreover, the modified DNAzymes suppressed the influenza virus expression in MDCK cells with high efficacy over 99% [65].

In another study, Kumar et al. prepared modified 10-23 DNAzymes, targeting various regions of the M2 gene and testing their ability to specifically cleave the target RNA [66]. The presented study described the cleavage activity of the designed DNAzymes both in in vitro and in cell culture experiments. Four DNAzymes targeted RNA to cleave at single stranded loop regions of M2 RNA. The researchers observed that only one DNAzyme showed a significant 70% downregulation of viral replication in MDCK cells compared to the others. Furthermore, the catalytic activity of DNAzyme was significantly correlated to the increasing concentration of DNAzyme [66].

Investigations described above indicated that catalytic nucleic acids could be a useful tool to control viral infection. The ribozymes and appropriately modified DNAzymes possess potent anti-influenza activity and can provide an opportunity to enhance the ASO strategy. 

In fast-evolving viruses, such as influenza, the development of effective therapeutics to limit disease spread is challenging. ASOs, siRNAs, miRNAs or catalytic nucleic acids tools afford the potential tractable strategies to eradicate the influenza virus. It is because they are easy to design, can be chemically modified to improve their stability and are directed against multiple virus strains by targeting the most conserved RNA regions. However, these tools act in a sequence-specific manner, which makes these approaches not highly effective in inhibiting the proliferation of influenza virus. Hence, the designing and development of new inhibitory oligonucleotides should be based on additional criteria using knowledge on the viral RNA secondary structure. 

### 1.2. Secondary Structure of Viral RNA and Its Importance for Rational Design of Oligonucleotide Therapeutic Tools 

As previously mentioned, influenza virus genome is organized into eight segments of single-stranded viral RNA. Each segment is composed of conserved, partially complementary 5′- and 3′-ends, segment-specific untranslated regions (UTRs) and open-reading frames (ORFs) [67]. During the viral life cycle, three different RNA types are produced: the vRNA is transcribed into mRNA and replicated through a cRNA intermediate into more copies of vRNA by the viral polymerase complex [6]. The vRNA and cRNA are shielded with viral nucleoprotein. Within a virus particle, vRNA is associated with the viral polymerase and assembled into a macromolecular vRNP complex. The correct packaging of all genomic segments into a single viral particle is suggested to be partially conferred by vRNA structural domains protruding from vRNP [68,69].

**Figure 1:**
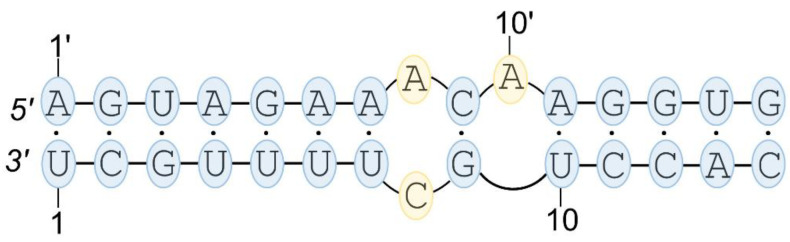
Scheme of the viral RNA (vRNA) promoter structure, called the panhandle motif.

The secondary structure of vRNA is an important regulator of virus biology, because it plays a role in assembling the different vRNPs into infective virions and in directing reassortments between IAV subtypes. The terminal fragments at the 5′- and 3′-ends of the vRNA segments (so-called panhandle structure, Figure 1) are highly conserved and known as the viral transcription and replication promoter. This region forms base pairs and results in a partial double-stranded structure which is bound by the viral polymerase. The rest of the vRNA (internal regions), via the phosphate backbone, is bound not uniformly by viral NP monomers and, with a stoichiometry of about 24 nucleotides per single nucleoprotein, forms a double-helical rod-like structure with part of the vRNA looped out and forming structural motifs [70,71]. In viral particles, the vRNP complex is a highly dynamic helical structure with the polymerase at one terminus and a closing loop at the other [72].

It is known that the panhandle structure is recognized by the viral polymerase [73]. Partial conformational change in the RNA structure induced by polymerase binding favors its promoter activity and results in the replication of viral mRNA [74]. Moreover, the long-range interactions of the panhandle structure are crucial for the regulation of influenza virus transcription and replication [75]. The panhandle structure is proposed to adopt three motifs depending on structural changes, i.e., the fork, the corkscrew and the hook [74]. The fork or corkscrew model has been proposed to explain the initiation of viral RNA transcription. It means that the viral polymerase is bound to double-stranded vRNA termini and the panhandle structure is partially relaxed, forming the fork or corkscrew motif in the promoter-polymerase open complex. In the hook model, the small hairpin at the vRNA 5′-end is necessary for polyadenylation of mRNA. Additionally, this structure seems to be important for binding the polymerase complex to the 5′-end of vRNA during mRNA synthesis [76]. Except for extensive investigations of the viral promoter structure and function, and a few analyses concerning the vRNA and mRNA structure, studies show that research into the structural properties of the influenza vRNA, cRNA and mRNA needs to be continued.

Some studies indicated that several short-range vRNA secondary structural motifs can play important roles in the influenza virus propagation. For instance, the Olsthoorn group predicted conserved RNA secondary structures in the NP segment of the IAV genome using structural analysis and comparative sequences. They identified structural elements, exhibiting nucleotide covariations and suggested the functional importance of a pseudoknot structure predicted in the NP packaging signal region [77]. Interestingly, several reports on the influenza virus virion structure indicate that multiple RNA interactions between the internal regions of vRNP can be involved in the specific packaging of eight vRNA segments [78,79]. In general, most segments could potentially interact with at least two neighboring vRNAs, and the fewest interactions occur between the vRNAs of the NA and NS segments. Interestingly, recent observations showed a direct interaction between viral genomic RNA segments of IAV and revealed that this contact takes place in infected cells and is important for the packaging of vRNA segments [80]. In particular, this study indicated that two vRNA hairpins from the PB1 and NP segments appear to combine via the complementary sequences of the loops, and together could initiate intermolecular contacts by forming a kissing loop structure [80]. More recently, Dadonaite et al. employed multiple high-throughput sequencing approaches to show distinct RNA conformations of different IAV genome segments and both intra- and intersegment RNA connections inside influenza virions [67]. They found that most IAV vRNA segments can interact with others, and there are many possible interactions inside the virions with different occurrence frequencies [67].

Importantly, several studies concerning the secondary structure models of the IAV genomic RNA segments have been conducted [9,10,81,82]. All these investigations allowed viral RNA structural features detection. The secondary structural motifs in the IAV naked genomic segments were determined using chemical structure mapping, thermodynamic energy minimization and structure-sequence conservation analysis. The obtained results revealed that proposed secondary structures for segments 8, 7 and 5 vRNA are preserved between IAV strains, which strongly indicates their importance in the viral life cycle. Moreover, these studies define structural elements universal in the type A influenza virus that might be new targets for potential anti-influenza therapeutics. In 2019, Incarnato and coworkers solved, for the first time, the in vivo secondary structure of IAV mRNAs in infected MDCK cells [11]. RNA structures probing with high-throughput sequencing allowed predicting of structural features of IAV mRNA transcriptome-wide. Interestingly, the researchers revealed that in vivo mRNAs are less structured in comparison with the in vitro refolded structure. However, they form specific stable conserved RNA motifs. Furthermore, they presented that the targeted disruption of these structural elements caused an attenuation of IAV replication [11]. These studies underline the importance of in vivo RNA structure prediction, and provide the means for future work aimed at targeting RNA with antiviral drugs. 

A detailed analysis of the viral RNA structure and information on RNA interactions vital for the virus replication cycle allow an understanding of the structure-function relationships, and can help the prediction of the influenza genomic regions that need to be taken into account during the designing and development of potential antiviral tools. Influenza RNA structure is often highly conserved across different type A strains, allowing to designate new targets for antiviral tools with high efficiency [83,84,85]. Additionally, further improvements in the prediction of conserved structural motifs and single-stranded regions (loops, bulges) may contribute to the rational identification of accessible and functionally important binding sites for antiviral oligonucleotide tools, which in turn raises their specificity and affinity. Specifically targeting conserved IAV RNA structural motifs represents a new opportunity to improve the effectiveness of antiviral drugs, especially due to the high genetic variability of the influenza virus.

### 1.3. Importance of RNA Secondary Structure in Designing of Anti-Influenza Therapeutics

**Figure 2:**
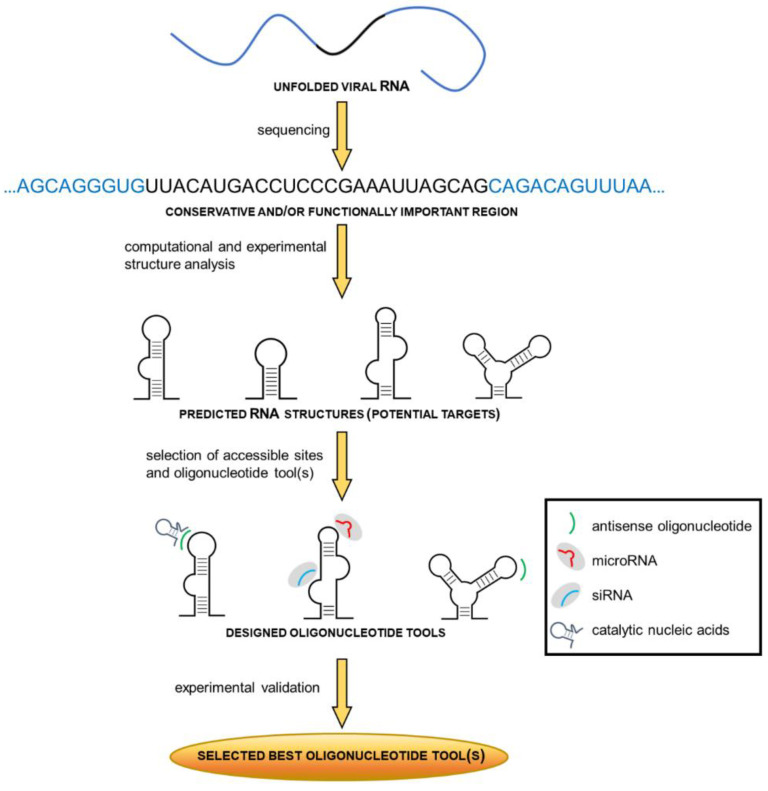
Scheme of the step-by-step strategy of oligonucleotide tools design and selection targeting influenza A virus (IAV) RNA.

Controlling of gene expression using chemically modified oligonucleotides as ASOs, siRNAs, miRNAs and catalytic nucleic acids has promising anti-influenza potential. Despite being investigated for many years, results of these therapeutic agents remain unpredictable, partially due to difficulties in finding effective target regions along the viral RNA. Since binding of oligonucleotides depends on thermodynamic properties and accessibility of desired sites for therapeutic agents, determination of RNA secondary structure seems to play a crucial role in developing a successful antisense strategy [86]. It seems that RNA structural motifs with long unpaired nucleotide tracks, such as internal loops and bulges, multibranch loops, long hairpin loops and long terminal single stranded regions, are favorable ASO targets [87]. The prediction of RNA secondary structure in combination with bioinformatics and experimental data enables the design of novel and highly bioactive therapeutics. This approach has been implemented and tested in influenza research (Figure 2).

Essentially, the conducted experiments have led to the discovery of internal single-stranded regions accessible for hybridization and structural motifs important for viral life cycle that were not previously targets of antisense strategies. In this part of the review, a structure-based target site selecting method, with an emphasis on its effectiveness, is presented. Throughout this section we will use the term ASO to refer to modified ASO with either 2′-O-methylated and/or locked nucleic acid (LNA) nucleotides.

In order to inhibit viral proliferation, Lenartowicz et al. used information about secondary structure of protein-free (in vitro) influenza A genomic segment 8 (vRNA) to design ASOs [19]. Prediction of the thermodynamics, base pairing and folding of vRNA segment 8 was supported by both comparative and experimental sequence analysis, including chemical and microarray mapping. Up to this study, mainly the panhandle region of influenza vRNA was targeted with ASOs. On the contrary, profound analyses of the secondary structure of IAV vRNA segment 8 made by Lenartowicz et al. revealed regions accessible to the ASOs outside the conserved 5′- and 3′-ends. Accordingly, oligonucleotides that were designed to bind experimentally confirmed the single-stranded internal regions. The most potent five ASOs caused inhibition of the virus replication, ranging from 5- to 25-fold in MDCK cells. It confirms that information about secondary structure might allow determination of novel and effective antiviral targets within influenza vRNA, which were previously considered inaccessible [19].

A similar strategy for selecting ASO-susceptible RNA regions for another influenza genome segment was employed by Michalak et al. [10]. A summary of information from computational sequence/structure analysis and structural mapping experiments not only provided the secondary structure of the entire vRNA segment 5, but also disclosed previously untargeted internal single-stranded regions. It was observed that five ASOs inhibited the virus replication from 1.8-fold to even 8.3-fold in MDCK cells [10]. High inhibitory result for the three most effective ASOs is probably related to their target sites accessibility, which is in concordance with the previously identified low NP in vivo binding profile [88]. These studies underline the effectiveness of in vitro viral RNA secondary structure prediction not only in designing ASOs, but also in discovering functionally important structural RNA motifs in influenza.

The significant role of secondary structure determination preceding the design of sites available for ASO applies not only to the genomic influenza segments, but also to (+)RNA. In the paper published by Soszynska-Jozwiak et al., influenza virus segment 5 (+)RNA secondary structure was established and validated by bioinformatics and experimental studies [9]. Nine of the most potent ASOs, which were designed to bind single-stranded, accessible internal regions, inhibit influenza replication from 3- up to 7-fold in MDCK cells [9]. An interesting issue addressed in this publication concerns oligonucleotides, which partially bind regions previously tested as effective ASO target sites in different influenza strains [34]. The antiviral effect confirmed by both authors is a plausible argument for the valuable contribution of viral RNA secondary structure determination in designing versatile ASOs targeting conserved motifs.

Overall, the results revealed above illustrate strong correlations between the predicted ASO binding regions and the ability to inhibit influenza virus replication. However, in each study some ASOs targeting single stranded regions exhibited moderate efficiency. This can be due to the dynamic nature of the structure of influenza RNA segments. Additional reasons, including local structure stability as well as interactions with other RNAs or proteins, also contribute to the antisense potency of ASO in the cells (in vivo). Nevertheless, besides playing an important role in designing efficient hybridization sites for ASOs, RNA secondary structure determination significantly contributes to understanding the influenza virus life cycle and replication mechanisms. The RNA structure-based target site selecting method provides novel prospects for other antiviral therapeutic agents such as small molecules.

According to several publications, the RNAi efficiency is influenced by the target RNA structure [89,90,91]. The target accessibility and the local alteration of its structure may have an inhibitory effect on target recognition and siRNA binding. Therefore, it is reasonable to consider it in the design of siRNA tools. Recently, Piasecka et al. described the studies concerning the IAV RNA secondary structure motifs as targets for siRNA-mediated RNA interference [92]. The researchers designed siRNAs targeting mRNA of segment 5 influenza virus using additional structural criteria for choosing the RNA target region. The siRNAs selected this way were effective against influenza virus type A at low concentrations. Moreover, the investigations led to the identification of regions which were accessible for RNAi machinery targets in the mRNA of segment 5, and as a consequence, significantly reduced viral replication of IAV [92]. Applying knowledge about viral RNA secondary structures as additional criteria in selecting siRNA molecules seems to be a promising approach.

Interesting research concerning the inhibition of influenza viral replication by targeting structural motifs of vRNA has also been conducted by Kesy et al. [93]. It is known that influenza viruses contain a conserved panhandle double-stranded RNA (dsRNA) region formed between the 5′- and 3′-termini of all genomic vRNA segments. The researchers reported that chemically modified dsRNA binding peptide nucleic acid (PNA) named dbPNA (dsRNA binding PNA) can bind to the panhandle motif and significantly reduced viral replication. They found that this chemically modified dbPNA oligomer selectively bound to the dsRNA region in the panhandle structure through a novel major-groove PNA-dsRNA triplex formation. Moreover, the dbPNA oligomer had a stronger binding affinity to its target compared with a single-stranded RNA (ssRNA) and a dsDNA containing the same sequence. In addition, the authors observed that the conjugation of dbPNA with an aminoglycoside called neamine enhanced the cellular uptake and, as a consequence, the viral protein expression was significantly reduced by the dbPNA-neamine conjugate targeting the panhandle structure [93]. The panhandle structural motif and other dsRNA regions of influenza virus genome are often rigid and may not be accessible to traditional antisense oligonucleotides; hence, novel dbPNA oligomers can be promising complementary tools for the viral inhibition or regulation of cellular functions.

Another structure-based approach uses the ability of small molecules to target specific RNA structures. In general, small molecules have the potential to recognize and bind to RNA targets based on their secondary or tertiary structures. Bottini et al. reported specific recognition of the vRNA promoter region (panhandle structure) by small molecules as a potential novel drug target and tool against IAV infection [94]. The authors identified 6,7-dimethoxy-2-(1-piperazinyl)-4-quinazolinamine (DPQ) and studied its inhibitory activity against influenza virus replication. The structural investigations revealed that DPQ binds to the RNA promoter in the major groove of its internal loop, preventing interaction between the promoter region (panhandle structure) and the polymerase complex. Inhibitory activity of DPQ may occur due to a structural change that interferes with the binding of polymerases subunits and subsequently the ability to replicate. Furthermore, DPQ analogues with modifications were prepared and their cellular activity against influenza was tested. The results demonstrated that most of these compounds showed improved inhibitory activities against influenza A virus in the micromolar range [94]. Based on these studies, it can be suggested that an RNA structure-based approach using small molecules provides a viable path to the development of anti-influenza potent inhibitors. The discovery of small molecule-targeting strategies directed at structured RNA motifs in the IAV genome should be continued. 

## 2. Conclusions

In summary, we presented examples of studies concerning ASOs, siRNAs, miRNAs and catalytic nucleic acids approaches as potential antiviral tools against IAV. We described investigations that focused on the viral RNA structure-function relationship and genomic conserved RNA regions as targets for oligonucleotides strategies. Moreover, we also discussed a new criterion for choosing and designing target sites in IAV segmented RNA that is based on information about its secondary structure.

Nowadays, given the large number of new imaging, sequencing and biochemical techniques, the influenza virus biology (i.e., genome structure and function) is relatively well understood. Recent advances in next-generation sequencing (NGS) technology provide new insights into the IAV genome architecture [95]. NGS techniques allow vRNA structure determination, including RNA-RNA or RNA-proteins interactions. Moreover, various types of high-throughput methods (e.g., coupled with crosslinking and immunoprecipitation) and electron microscopy are utilized to study the assembly of the influenza virus [95]. Due to the development of the abovementioned techniques and bioinformatics tools, researchers are able to determine the vRNA structure and the conserved motifs. As a consequence, it is possible to design oligonucleotide-based strategies targeting the IAV vRNA with high specificity. 

However, an efficient oligonucleotide tools delivery to the target cell still remains a challenge. To promote the intracellular uptake, many effective approaches are being developed, including oligonucleotide chemical modifications, bioconjugation of various delivery-promoting moieties or usage of nanocarriers [96]. In addition, the disadvantages of oligonucleotide-based anti-IAV approach involve inefficient binding to the target site and low effectiveness. To overcome these obstacles, two or more types of oligonucleotide tools can be combined, for instance, ribozyme with antisense molecules [61]. Furthermore, the combination of various antivirals could reduce the probability of drug resistance emergence. Roosens and co-workers described that NGS technology can be used to monitor and detect antiviral resistance mutations within the IAV genome [97]. This approach may be useful in surveillance and development of any new class of oligonucleotide-based anti-IAV drugs. 

In the last few years, the CRISPR-Cas system has become one of the most powerful gene editing tools. Interestingly, several studies using a CRISPR-Cas13-based strategy have been performed on potential inhibition of RNA viruses, including IAV [98] and the newly emerged severe acute respiratory syndrome coronavirus 2 (SARS-CoV-2). However, CRISPR-Cas technology still raises concerns about off-targets effects; additionally, its delivery method efficiency needs to be evaluated [99]. Despite that the CRISPR-Cas system-based antiviral strategy requires further research, it seems that it will soon lead to the discovery of various promising candidates for IAV treatment. 

In light of the continuous evolvement of IAV and its increasing resistance to existing drugs and vaccines, finding target sites in IAV RNA that are sequentially or structurally conserved and less prone to drug resistance is a very important issue. That conclusion could be extended to other RNA viruses, including SARS-CoV-2. A novel therapy concerning the regulation of pathogenic RNA by using RNA-targeting strategies is still being developed. The flexible and dynamic nature of viral RNA structures is a challenge associated with targeting RNAs with potential therapeutics. Therefore, information regarding the molecular and structural basis behind influenza virus biology, including knowledge about RNA secondary structure, could be used to design and develop structure-based approaches that will inhibit influenza replication. The ability to identify oligonucleotides or molecules that specifically bind to RNA targets on the basis of its structure provides a viable path to create new drugs against influenza.
